# Electrosurgery: heating, sparking and electrical arcs

**DOI:** 10.52054/FVVO.16.3.026

**Published:** 2024-09-30

**Authors:** PR Koninckx, A Ussia, B Amro, M Prantner, J Keckstein, S Keckstein, L Adamyan, A Wattiez, A Romeo

**Affiliations:** Prof. emeritus Obstetrics and Gynecology KULeuven, Leuven, Belgium, the University of Oxford, Oxford, UK, Università Cattolica, Rome, Italy and Moscow State University, Moscow, Russia; Gruppo Italo Belga, Villa del Rosario, Rome Italy; Latifa Hospital Dubai, UAE; BOWA-electronic GmbH & Co. KG, 72810 Gomaringen, Germany; Endometriosis Centre, Dres. Keckstein, 9500 Villach, Austria; Faculty of Medicine, University Ulm, 89081 Ulm, Germany; Department of Obstetrics and Gynecology, LMU University Hospital, Munich, Germany; Department of Operative Gynecology, Federal State Budget Institution V. I. Kulakov Research Centre for Obstetrics, Gynecology, and Perinatology, Ministry of Health of the Russian Federation, and Department of Reproductive Medicine and Surgery, Moscow State University of Medicine and Dentistry, Moscow, Russia; University of Strasbourg, Strasbourg, France; Research Educational Center, University of Turin, 10124 Turin, Italy

**Keywords:** Laparoscopic surgery, electrosurgery

## Abstract

The translation of impedance (R), current (I), and voltage (V) into tissue effects and the understanding of the settings of electrosurgical units is not obvious if judged by the many questions during live surgery. Below 200 V, the current heats the tissue until the steam of boiling stops the current. Thus, slower heating, because of less energy or a larger contact area, results in deeper coagulation. Above 200 V and a duty cycle (per cent of time electricity is delivered) of >50% (yellow pedal), sparks become electric arcs, and the heat causes the explosion of superficial cells, i.e. cutting. With higher voltages, cutting is associated with coagulation, i.e. blended current. With even higher voltages and a duty cycle <10% preventing arching, only coagulation occurs (blue pedal; forced coagulation).

Voltage being crucially important for tissue effects, newer electrosurgical units deliver a constant voltage and limit the energy output (Maximal Watts: W=I*V= joules/sec). Unfortunately, the electrosurgical units indicate the combination of voltage and duty cycles as a force of cutting (pure cutting or blended) or coagulation (soft, forced or spray) current. It is important that the surgeon understands whether electrosurgical units control voltages or output, as well as the electrical basics of the different settings and programs used.

## Introduction

Surgery combines knowledge of anatomy with skills such as cutting, dissection, bleeding control, and suturing. Massarweh et al (2006) nicely reviewed the history of electrosurgery. The clinical use started with the introduction of Bovie’s first electrosurgical unit in the early 1920s (Bovie, 1995). Other energy sources, such as lasers, ultracision and plasma jets, were added later.

Electrosurgery is governed by the laws of electricity describing the relationship between voltage(V), current (I), ohmic resistance of tissue (R) and heat delivered. These are Ohm’s law (I=V/R), Watt’s law (power or Watt=IV=I2*R), Joule’s law (heat delivered=W*t or I2*R*t) and Coulomb’s law, describing the force between two electrically charged particles and the distance. This Coulomb force generates sparks when a sufficiently high electric field creates an ionised, electrically conductive channel through a normally insulating medium such as air or CO2. A spark becomes an electric arc if the energy supply is sufficient to maintain a sufficient voltage, notwithstanding the lower impedance. Electrosurgery requires a high- frequency alternating current of more than 100,000 Hertz to prevent galvanic and Faradic effects with depolarisation of cellular membranes, muscular contractions or nerve stimulation as occurs with 50Hrz of domestic electricity. The electrosurgical units have improved over time by overcoming technical difficulties and introducing voltage stabilisation or adding safety measures such as separate high-frequency generators for coagulation and cutting or ‘intelligence’ adapting voltage or output by measuring tissue impedance (e.g. PK devices) or compression and temperature (sealing devices).

It can be confusing for the surgeon to translate V, I, R, and W into electrosurgical unit settings and surgery requirements, such as safety, tissue damage, speed and depth of cutting and coagulation. Depth of tissue damage can be critical in ovarian and bowel surgery, knowing that the colon wall measures only 1.5 to 3 mm (Farin and Grund, 2009). Also, although well-known, capacitive coupling is poorly intuitive and has new potential risks, such as the risks of electrical cables running together and during port laparoscopy (Townsend et al., 2017).

The terminology of electrosurgery is confusing and not precise (Morris et al., 2009). Although tissue damage begins with unwinding the 3D structure or denaturation of proteins, coagulation describes three effects: the reorganisation of proteins, the transformation of collagen into a coagulum, and the desiccation of tissue becoming electrically more resistant. The waveform of the alternating currents in electrosurgery is always sinusoidal, which can differ only in voltage and duty cycle or per cent of time energy is delivered. Cutting means > 200V and a duty cycle of more than 10%, generally more than 50%. Forced coagulation uses very high voltages and a less than 6% duty cycle to prevent arcing (ASGE Technology Committee et al., 2013). Therefore, words such as cutting wave, blended wave, or coagulating wave, often used in the literature and during live surgery, are misleading since they suggest specific waveforms. Radiofrequency (Munro, 2012) can be used to indicate that the frequencies of the alternating current used in electrosurgery are similar to those of radio waves. However, radio waves are electromagnetic waves used in diathermy, which is confusing. Radiosurgery (2 MHz) and radiocautery (3-4 MHz) only indicate frequency differences (Holmstrom et al., 2004). Impedance and resistance are frequently used interchangeably. The impedance is composed of the ohmic resistance and the reactance. The reactance depends on the frequency and on the inductances and capacitances of the electric circuit. The intensity of the current flowing within tissue depends on the impedance of the tissue, whereas the heat generated by this specific current solely depends on the ohmic tissue resistance. Hence, impedance should be used for alternating currents with voltage changing over time, and resistance should be used with respect to heat generation. Capacitive coupling is occasionally called stray energy or parasitic capacitance (Wikiel et al., 2023).

Understanding electrosurgery is important for the surgeon (Vilos and Rajakumar, 2013), but knowledge is often limited in gynaecology (Pandey et al., 2007). Therefore, we reviewed the fundamentals of electrosurgery and tissue effects for gynaecologic surgeons. A discussion of other energies, such as lasers and ultracision, and their indications of use, is beyond the scope of this manuscript.

## Electrosurgical Basics

**Figure 1:**
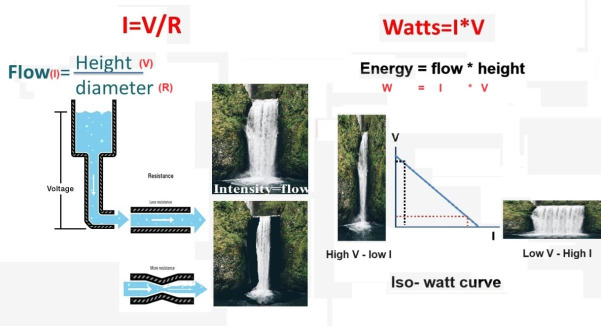
The basic laws of electricity I= V/R or Ohm’s law, and W=I*V or Watt’s law. This can be compared to water flow (current intensity, I) increasing with the height or pressure (voltage, V) and decreasing because of a narrowing (impedance or resistance, R). Thus, the same energy can be obtained with little water falling from higher or more water falling from lower. In electricity, this is called the isowatt curve.

Electrosurgery uses an alternating current with a frequency of more than 100,000 Hertz, generally between 250,000 Hz and 4 MHz, to avoid depolarisation of cell membranes, muscle contractions or nerve stimulation. The voltage of an alternating current is defined as the peak or highest voltage. Electricity follows Ohm’s law, I= V/R, describing the relationship between current intensity, increasing with voltage and decreasing with impedance. The tissue impedance varies with its water content but is low in gynaecological or abdominal surgery, except for fat tissue. Therefore, the current varies almost exclusively with the voltage and the contact area of the instrument with the tissue (Figure 1).

According to Watt’s law, the power (Watts, W) delivered (W=I*V = I2R), increases with the current intensity and the voltage or the second power of the intensity times ohmic resistance (V=I*R). Thus, the same amount of energy (power/sec) can be delivered with a low voltage and high current or vice-versa, the so-called iso-Watt curve.

**Figure 2:**
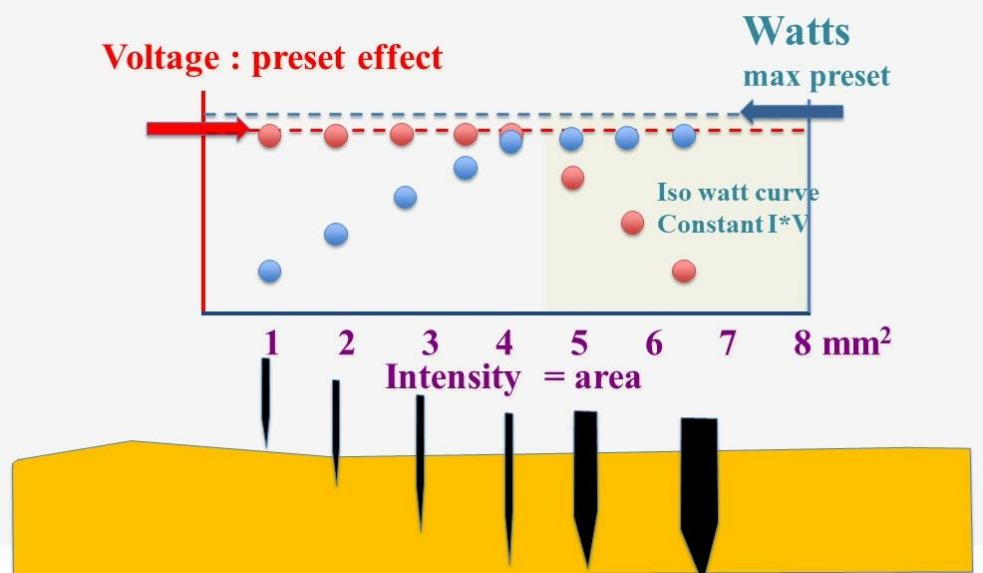
The electrosurgery unit’s maximal output (max Watts) and the voltage and delivery cycle are preset values. The current intensity (blue dots) is minimal when the contact area is small and increases with the contact area, but with a voltage stabiliser, the voltage (red dots) remains constant (left white area). However, when the contact area and I increase further, and I*V exceeds the preset maximal output, the voltage has to decrease according to the isowatt curve (right yellow area).

More recent electrosurgical units with a voltage stabiliser permit limiting the maximal output (max W) and setting the ‘force’, which defines the voltage and the duty cycle, often called current modulation. Unfortunately, manufacturers indicate this differently and not very transparently. For a given force setting, the current varies only with the contact area of the surgical instrument with the tissue and the water content of tissues, being low in gynaecological surgery except for bone or fat tissue. Thus, the current intensity will be low when the contact area is small, such as a needle electrode, and only a tiny part of the available power (Watt) will be used. The current intensity increases when the contact area is larger, till the preset maximal output (max W) is reached. When the contact area increases further, the current Intensity rises further, but the voltage has to drop when V*I exceeds the preset max W output (Figure 2).

The energy delivered varies, besides V and I (W=I*V), with the duty cycle, which is the percentage of time measured in milliseconds that the energy is delivered. Thus, a continuous current intensity delivers the same energy as a current intensity with twice the voltage during half the time. The duty cycle is fundamental in electrosurgery, although often misleadingly described as an altered waveform, which is always sinusoidal.

**Figure 3:**
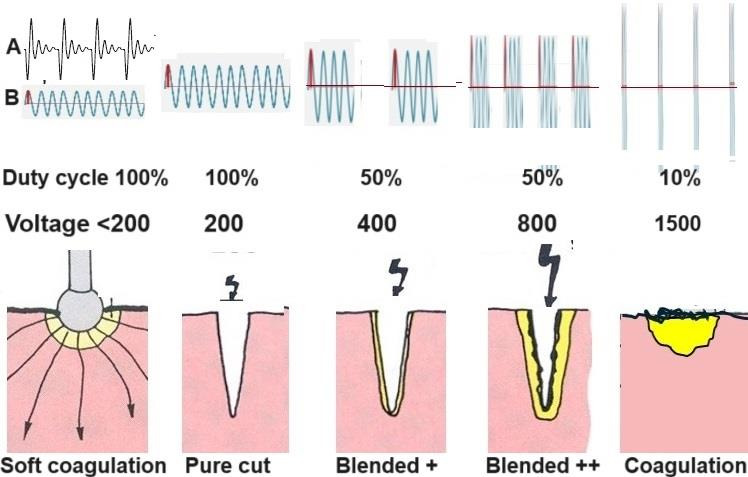
Tissue effects vary with voltage or crest factor and duty cycle. Today, a high-frequency alternating current has a sinusoidal waveform with a constant peak voltage (B). Historically, the current was consecutive bursts of higher voltages that decreased rapidly, with the crest factor being the ratio of the peak over the average voltage (A). less than 200V results in soft coagulation. Above 200 V and a duty cycle >50%, sparking becomes arcing, causing vaporisation and cutting. The thermal damage of the edges is only 2µ at 200V (Palanker et al., 2008b), but coagulation of the edges increases with the voltage (blended current). The middle picture illustrates that a double voltage and a duty cycle of 50% deliver the same energy but with more coagulation. Forced coagulation uses the coagulation effect of high voltage sparks without arcing because of a duty cycle of less than 10%.

Sparks are abrupt electric discharges occurring when the electric Coulomb field exceeds the insulating power of air or CO2, creating an ionised, electrically conductive channel (Figure 3). A spark is facilitated by a higher voltage, a shorter distance between the tissue and a sharp edge or a point electrode. With sufficient energy supply (W) and a more than 50% duty cycle, a spark can become an electric arc. Since the R of an arc is low, electric arcs require a high current intensity, although the voltage of an arc can be slightly lower than the voltage needed for initiation. Translated to electrosurgery, spark initiation is non-contact, requires a voltage higher than 200V (or >400V peak to peak) (Palanker et al., 2008a), and is facilitated by a short distance and an instrument with a sharp edge or a point. Avoiding contact between the electrode and the tissue is crucial to maintain the electric arc.

Electrosurgery devices only indicate the tissue effect, such as cutting or coagulation, activated by the yellow or blue pedal, respectively, and a qualification, such as 1 to 4 force, blended, or a program. Voltages are rarely indicated for many reasons. Historically, voltages were not constant since high-frequency electrosurgical currents were obtained mechanically with capacitance coupling, resulting in short bursts of high voltages that decreased rapidly over time. Voltages between instruments and tissues vary with the current intensity because of the device’s internal impedance. However, the main reason for not indicating voltages is the importance of combining voltages and duty cycle. Cutting current requires arcing and higher voltages with a duty cycle of more than 50%; for coagulation, arcing is avoided by soft(slow) coagulation with less than 200 V or faster (forced) coagulation with very high voltages but less than 10% duty cycle.

Electrosurgery can be used in monopolar or bipolar mode. In monopolar mode, the electricity flows between the active electrode and a large return plate with a low electrical density. In bipolar mode, the electricity flows between the two tips of the instrument.

## Tissue effects

The tissue effects of electrosurgery result from heating tissue. Tissue damage by temperatures up to 44°C is reversible, but after that, irreversible denaturation of proteins, although slow, increases with temperature and becomes immediate above 80°C. At 100°C, water starts boiling with minor but surgically irrelevant variations caused by pressure or electrolytes. Slow boiling causes desiccation by evaporating water, leaving minerals and tissue debris. Very fast increasing temperatures cause all water to transform suddenly into steam, causing an explosion of cells or vaporisation, containing tissue debris with possibly a health risk of the smoke with eventual viruses (Hurst and Stewart, 2024). Above 300°C, proteins and nucleic acids start to oxidise, combining the hydrogen or organic molecules with the oxygen in the air and leaving the carbon, called charring. Poorly understood is the unfolding and recombination of collagen fibres, occurring when the pressure on the tissues is sufficiently high and the temperature is strictly regulated at around 80°C.

## Electrosurgical coagulation

**Figure 4:**
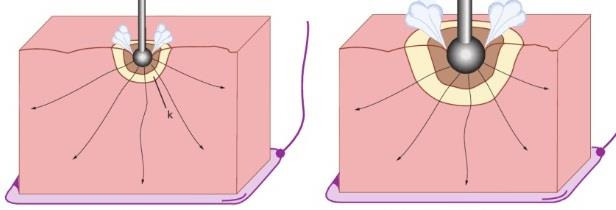
The depth of coagulation increases with a longer duration of heating and more heat diffusion before the current is stopped by steam development. Deeper coagulation thus occurs when the contact area is larger.

Coagulation and desiccation occur when tissue is heated progressively without sparks. When the active electrode, monopolar or bipolar, is in contact with the tissue, the energy delivered, or tissue heating (W=R*I2) is proportional to the tissue’s impedance (low but much higher than the impedance of the electrical circuit) and the second power of the current intensity. With monopolar coagulation, heating occurs mainly in the tissue in close contact with the active electrode, since the current density (current intensity/diameter or volume) decreases to the third power with the distance from the electrode. The electric current intensity heats the tissue until the superficial layer reaches 100°C, and steam development stops the electrical current by isolating the tissue from the electrode. A clinical consequence is that slower coagulation is deeper coagulation since slower heating permits deeper heat diffusion. A larger contact area increases the coagulation depth for the same reasons since a larger tissue volume is heated (Figure 4).

With bipolar coagulation, the amount of tissue between the jaws is limited, the distance is small, and the current flow is parallel, resulting in more homogenous tissue heating and desiccation before the increased impedance of desiccated tissue stops the current. The bloodstream of an eventual artery needs to be interrupted by pressure or rotation to prevent local cooling. Measuring the temperature under the electrode or the tissue’s impedance permits adaptation of the current, preventing steam development until complete desiccation. Sealing devices take advantage of the reorganisation and fusion of collagen when the pressure between the jaws is sufficiently high, and the temperature is strictly controlled at around 80°C. Fat has a high impedance and can be used as isolating material. Therefore, sealing devices are more suited for fat tissue, such as omentum or bowel surgery, than classical electrosurgery.

Soft coagulation occurs when the voltage is below 200V without sparks. Unfortunately, heat delivery is slow, resulting in slower coagulation, which is impractical when coagulation areas are large or cooled by bleeding. This might be useful when a fragile vein needs to be coagulated slowly. Soft coagulation is recommended for bipolar scissors since less damaging because of the absence of sparks. When the max output is limited, the surgeon can use the iso-watt curve by taking a big bite of tissue, thus increasing the contact area and current intensity, and decreasing the V when I*V exceeds the preset output (Figure 2 (right)). However, the surgical challenge is often faster coagulation without cutting. This requires very high voltages up to 5000 V without arcing because of a duty cycle of less than 10%, generally <6%. The surgeon can also use repetitive bursts of coagulating current to prevent arcing when the impedance increases because of desiccation and can also avoid arcing by cooling the coagulation area with irrigation.

Effective coagulation thus results from a combination of the device settings and the surgeon. When pressing the blue pedal for coagulation, the electrosurgery device facilitates fast coagulation with very high voltages but without electric arcs because of a very low-duty cycle. Since the maximal energy output is limited, the surgeon can take advantage of slightly lower voltages and longer duty cycles combined with large contact areas and high intensities and with intermittent coagulation without full desiccation or active cooling.

## Electrosurgical cutting

Electrosurgical cutting occurs because of the heat of electrical arcs (>1000°C), which causes an explosion of the superficial cells or vaporisation. A higher voltage, a shorter distance between the instrument and the tissue and a sharper edge or a point, facilitates a spark. The distance to initiate a spark is slightly shorter in a CO2 environment than in air, but this is clinically insignificant (Culp et al., 2016). The spark can become an electrical arc with a low impedance if the energy supply (W) is sufficient and the duty cycle is at least 50%. The tissue effects vary with the voltage over the arc. An arc of 200 V results in cutting with minimal thermal damage to the edges (as little as 2µ, (Palanker et al., 2008b)), but when the voltages are higher, the thermal damage of the edges increases and causes increasing coagulation (Figure 3). This combination of cutting and coagulation is called blended current. Unfortunately, it is counter-intuitive that even higher voltages with a duty cycle of less than 10%, insufficient to maintain an arc, are called coagulating currents.

Thus, electrosurgical cutting is caused by continuous arc, vaporising tissues. With higher voltages, cutting is increasingly associated with coagulation, called blended current. Using the point of a needle electrode, e.g. in microsurgery, with only one arc, surgery at exactly 200V with minimal lateral damage can be undertaken. However, larger instruments, such as the electrical bistoury, generate a series of sparks and arcs along their cutting edge, and the current intensity varies with the contact area and the cutting speed (Bao and Mazumder, 2023). However, even with a voltage stabiliser, it can be difficult to maintain 200V arcs because small current intensity fluctuations can exceed the preset maximal output.

Electrosurgical cutting is non-contact surgery for the surgeon, which is to be activated before contact because of sparking and arcing. The surgeon determines the speed and depth of cutting, which are limited by the maximal output. The blend effect increases with the voltage over the arc, from 2µ (Palanker et al., 2008b) at 200 V and becomes important with higher voltages (Figure 3). Clinically, fat tissue is less suited for electrosurgery because of its higher capacitance and low water content.

## Comments on electrosurgical coagulation and cutting during laparoscopic surgery

More recent electrosurgical units can modulate the output and voltage according to the desired effect when tissue impedance varies, such as in gallbladder surgery. In gynaecological surgery, impedance variations are limited to fat tissue and the degree of desiccation. This is used in bipolar coagulation to increase the voltage and keep the same coagulation speed when the impedance increases because of desiccation. Cutting and coagulating currents can also alternate to obtain the desired effect as used in Endocut mode (ASGE Technology Committee et al., 2013). Since stabilising voltage is technically difficult, older electrosurgery units keep the output constant (Friedrichs et al., 2012) to facilitate electrosurgery when tissue impedance varies. Clinically, it is important to understand whether the electrosurgery unit has a constant output or a voltage stabiliser. Both are often easily recognised by their output indication as Watts or Maximal Watts.

Bipolar coagulation is localised between the two tips, permitting underwater coagulation (Ushimaru et al., 2022). Clinically, this can be used for pinpoint coagulation by limiting the lateral spread of heat and damage by continuous irrigation, as we used to do in microsurgery. Short bursts of coagulation with irrigation with a pointed bipolar instrument keep their full importance when coagulating bleeding from the ovary to prevent oocyte damage or from the rectum during deep endometriosis excision, considering the thin rectum wall. The coagulating sparks without arcing between the bipolar and peritoneum without making contact can weaken the peritoneum locally, which can subsequently be entered with simple pressure.

With a voltage stabiliser, cutting thin tissues, such as the peritoneum, is independent of the preset watts since only a limited amount of the available maximal output is used, as illustrated in Figure 2 (left). The maximal output (Watts) only determines the area that can be sectioned and the movement speed without dropping the voltage. The preset maximal output is mainly a safety feature, limiting accidents by dropping the voltage when, during inadvertent movements, the contact area becomes large. For example, a 30-watt output permits cutting 0.5 cm of tissue but prevents accidentally cutting a bowel over 2 cm. A limited maximal output lets the surgeon drop the voltage by increasing the contact area.

Measuring the impedance and temperature of tissue surface and the pressure between the jaws of a bipolar can be used to optimise coagulation or for specific effects such as collagen fusion or sealing. Boiling and steam development can be prevented by interrupting the current permitting cooling. An acute increase in impedance indicates desiccation (PK devices). Sufficient tissue compression, combined with a temperature around 80°C, results in collagen reorganisation and fusion, as used in sealing devices. This permits the securement of larger vessels and the coagulation of fat tissue.

Sparking and arcing vary with the gas environment, as used in the argon plasma coagulator, which uses longer sparks to control larger areas of diffuse bleeding, such as during liver surgery.

## Electrosurgical safety

Monopolar electrosurgery requires a return plate with a large contact area attached over a well- irrigated muscle mass without areas of higher impedance, such as the knee, between the active electrode and the plate. Care should be taken when pacemakers or other implantable devices with lead wires are present, and prosthetic conductive joints such as hip replacements should be avoided (Massarweh et al., 2006). The impedance between the two halves can be monitored as a safety measure to detect partially detached grounding plates. The risk of isolation failure increases with the high voltages for cutting with coagulation or forced coagulation. Unfortunately, isolation failure occurs in 20% of reusable instruments (Montero et al., 2010) and cannot be detected by visual inspection (Tixier et al., 2016). Direct coupling permits the transmission of electric current from one instrument to another, as done in open surgery and in laparoscopic surgery before the grasping bipolar forceps became available.

Capacitive coupling is the well-known transmission of an alternating electric current without direct contact using two opposing metal plates: when one plate becomes positive or negative, the other becomes negative or positive, respectively. A well-known example of capacitive coupling during laparoscopic surgery is when a metal trocar, insulated from the abdominal wall with a plastic screw, picks up the alternating current from an inserted instrument and causes thermal damage. Poorly studied is the risk when two instruments are in close vicinity over a longer distance, such as during single port surgery (Brinkmann et al., 2022). Similarly, heating by up to 40°C was observed by energy transfer between the cables from the monopolar instrument, the return pad, and other wires (Townsend et al., 2016). Also, the risk of capacitive coupling between the tips of 2 instruments during robotic surgery should be realised (Abu-Rafea et al., 2011; Mendez- Probst et al., 2011; Wikiel et al., 2023). Finally, electrosurgical instruments can remain hot after being used, potentially damaging other tissues.

## Discussion

Besides Ohm’s (I=V/R) and Watt’s (W=I*V) law, electrosurgery requires understanding Coulomb’s law regulating sparking and electrical arcs with a lower impedance and a continuous current. Arcs require a more than 50% duty cycle and sufficient energy supply. Whatever the settings, the surgery continuously changes V, I, and R. In wet tissues, the current intensity increases with the contact area, and V decreases if V*I exceeds the maximal output. During desiccation, the impedance increases, and I decreases. The conductive ionised channel of arcing has a low impedance, increasing the current intensity. Therefore, the electrosurgical unit controls either the voltage or the output, or both besides a maximal energy output. A voltage stabiliser emphasises a constant tissue effect, and the current intensity varies with the impedance or contact area up to the preset maximal production. For specific purposes such as loop coagulation of intestinal polyps, keeping the output constant or using alternating cutting and coagulating current can be helpful.

Electrosurgery’s tissue effects result from the water temperature causing protein denaturation, unfolding and reorganisation of collagen (sealing) at 80°C (and pressure) and boiling at 100°C. Slow boiling causes desiccation. When all the water suddenly transforms into a vapour it is similar to an explosion. An electrical arc of 200V causes cutting with minimal damage or coagulation, but with increasing voltages, the depth of coagulation increases. Cutting with coagulation is called blended current as in older electrosurgical units such as the Bovie, the high frequency alternating current was a sequence of bursts of high voltages decreasing rapidly, and thus a mixture of high and lower voltages. Forced coagulation uses the coagulating effect of high voltages up to 5000V without arcs because of a low duty cycle of around 6%.

The surgeon should understand the basics of the electrosurgery unit settings. The Watt or energy output is essential in output-controlled units. In units with a voltage stabiliser, the maximal output is a safety feature, and it permits a voltage drop when exceeded by I*V because of a high I due to a larger contact area. To understand tissue effect, voltages and duty cycles are fundamental, but electrosurgical units only indicate for cutting (=arcing) the blend effect of higher voltages as forces or blend 1 to 4. Coagulation is indicated as soft (<200V), forced (very high voltages and a low duty cycle preventing arcing), or spray (even higher voltages) coagulation. Today, the combination of voltages, duty cycles, and tissue effects can be more complex when regulated electronically by specific programs. The details of electrosurgical unit settings also vary between manufacturers. It helps to understand that higher voltages during arcing facilitate cutting since distance is less crucial. It is nice to know that higher arcing voltages often have a duty cycle of only 50% to prevent exceeding the preset maximal output. The preset maximal output determines the amount of tissue that can be cut, i.e. the depth and the speed of movement. Deeper soft coagulation or desiccation requires slower coagulation to prevent steam development to stop the current. The surgeon should know that cutting is non-contact surgery requiring activation before making contact and that coagulation is contact surgery requiring activation of the current after contact of the electrode with the tissue. However, tissue effects of coagulation with very high voltages without electrical arcs because of a low-duty cycle and cutting with high voltages and arcs are similar, as demonstrated by the depth of tissue damage being similar when removing intestinal polyps with snare technology (Pohl et al., 2020). The depth of coagulation and lateral heat damage, being critical in ovarian and bowel surgery, is minimal, with a pointed bipolar as used in microsurgery and short coagulation. The lateral spread (Stefanovic et al., 2023) and coagulation depth were 1.1 and 1.3 mm after 2 or 4 sec of bipolar coagulation (Siracusano et al., 2023). It should be understood that a bipolar grasping forceps for laparoscopic surgery is a compromise between grasping strength and a small tip to permit pinpoint coagulation. Plasma jets or plasma scalpels (Xiao et al., 2022) cannot result in pinpoint coagulation. Most importantly, understanding electrosurgery will help the surgeon find an equilibrium between settings, the technique of surgery, and the choice of instruments since changing electrosurgery settings during surgery is impractical. The selection of using the tip of scissors, a needle, or a hook for electrosurgical cutting varies with experience and personal preferences and not because of underlying electricity rules.

New developments include advanced sealing devices (Brill, 2008), although the molecular nature of the sealing mechanism and the induced tissue effects remains poorly understood (Kramer and Rentschler, 2018). Other energy sources, such as CO2 or diode lasers and ultrasonic devices and even newer developments such as deep learning and artificial intelligence (Han et al., 2019) or the introduction of acoustic signals signalling instrument tissue interaction (Ostler et al., 2020) are beyond the scope of this manuscript.

## Conclusions

Electrosurgery is a versatile energy source during surgery, permitting dissection, cutting and coagulation as summarised in Table I. Duty cycles, voltages, sparking, and arcing are important for understanding tissue effects but are poorly indicated on electrosurgical units. Also, the non- precise terminology used in electrosurgery risks causing confusion among surgeons.

**Table I:** Electrosurgery for the clinician.

Ohm’s law: I=V/R or current intensity=Voltage (or power)/Impedance (the resistance of alternating current).
Impedance is low for wet tissues. Current intensity thus increases with V and contact area The current intensity increases with a larger contact area. When I*V exceeds the preset maximal output (Watts), The Voltage has to drop Impedance is high for dry tissue (desiccated), fat and air (vapour of boiling stops coagulation).
Voltage, sparking and electrical arcs
Sparking is facilitated by higher Voltages (>200V), shorter distances and sharp edges. With sufficient continuous (duty cycle >20%) energy (I*V) supply, a sparking can become an electrical arc. An electrical arc is a current through a conductive channel of a thermally ionised column of gas, such as air, called a plasma. An arc emits light and has temperatures up to 2500° C.
Cutting: the high temperature of an electrical arc vaporises superficial tissues.
An arc of 200 V is pure cutting. Higher voltages (up to >1000 V) cause coagulation besides cutting (blended current).
Coagulation
Soft coagulation heats and desiccates tissues without sparks, i.e. < 200V. Slower coagulation is deeper coagulation. Coagulation: >2000 V with a duty cycle of <10% uses the coagulation effect of high voltages but without an arc. Spray coagulation: the larger areas of non-focused Intermittent arcs over larger distances using specific gases are used for superficial coagulation by heating. Tip for Pin-point superficial coagulation: a pointed bipolar + short bursts of electricity (2-3 sec) + continuous cooling by irrigation.
